# Notes and News

**Published:** 1900-04-21

**Authors:** 


### Notes and News.

HOSPITAL MEETINGS.

St. George's Hospital.—Quarterly Special Court at one o'clock p.m., on Friday, April 20th.

London Female Lock Hospital.—Annual Meeting at five o'clock p.m., at 91, Dean Street, Soho, on Thursday, April 26th.

The new workhouse infirmary for Winchester has been opened. The building is not a new one, having been originally the fever hospital. The alterations and additions which have been made to render the infirmary thoroughly suitable and efficient have been secured at a cost of £50 per bed, so that the institution has been obtained by the Guardians in a very economical manner.

The new small-pox hospital for Guildford, Godalming, and Woking, on Whitmoor Common, has been opened recently. It is to contain six male and four female patients, located in a good-sized pavilion. The administrative block is separate, and there is also laundry, mortuary, and porter's lodge. The drainage of refuse water is passed through a coke filter into land especially prepared for the purpose. Earth closets will be used exclusively. The water supply will be supplied by the Woking Company. The cost of the building was £5,300, and that of furnishing £200.

The recently-issued report by Dr. Joseph Priestly as to the work done in connection with the bacteriological laboratory established by the Lambeth Vestry shows how greatly such an institution may, if in good hands, assist in tracing epidemics and in maintaining a high standard of sanitation. Among other things some very interesting observations were made as to the existence of diphtheria baccilli in the slime lining the open troughs leading from lavatory basins in a school in which, after a long prevalence of infectious sore throats, true diphtheria had at last broken out. The differentiation, by aid of the Widal reaction, of mild cases of typhoid from among a more widely spread outbreak of influenza in a poor district was another example of well-applied bacteriological work for public health purposes.

The new fever hospital for Perthshire at Burghmuir, Perth, has been opened by the Vice-Convener, Mr. A. Hutcheson. It is to contain 32 adult patients, and is constructed of wood and iron, at a cost of £100 per bed.

In our note last week in reference to the London School of Tropical Medicine an error was made in the date of its opening. This should have been October 2nd, 1899, not 1898, as stated. In the six months since that date 52 students have joined.

The London County Council has a scheme for building houses for the working-classes at Tooting. The District Board of Works of Wandsworth and the Streatham and Tooting Local Committee have inquired into the matter, and protest against the alleged intention to place about thirty houses on an acre of ground, thus creating a worse density of population than in the metropolis itself. Further, they protest that the charges will be higher than those of the present local tenements, whilst the children will have no playground but the streets.

The verdict of temporary insanity in cases of suicide has long since become a mere meaningless formula, which, although perhaps comforting to relatives and trouble-saving to jurymen, bears generally but little relation to the facts of the case. How entirely without meaning the formula has become, and to what a mere routine inquests are sometimes reduced, may be judged from the report in one of the Surrey papers of an inquest recent held on one of the inmates of the Brookwood Asylum who had strangled himself. As usual, a verdict of "suicide whilst temporarily insane" was returned.

At the Evelina Hospital for Sick Children the following post-graduate course of clinical demonstrations on diseases of children will be given on Wednesdays at half-past four p.m.: Medical cases, Dr. Nestor Tirard, May 9th; surgical cases, Mr. A. H. Tubby, May 16th; ophthalmic cases, Mr. Sydney H. A. Stephenson, May 23rd; medical cases, Dr. Fred Willcocks, May 30th; surgical cases, Mr. F. C. Abbott, June 6th; dental cases, Mr. Denison Pedley, June 13th; medical cases, Dr. George Carpenter, June 20th; surgical cases, Mr. Walter Edmunds, June 27th; medical cases, Dr. W. Soltau Fenwick, July 4th; surgical cases, Mr. C. H. Fagge, July 11th. The fee for the course of ten demonstrations is one guinea.

The New York *Medical Record* describes a new disinfecting steamer which is about to be sent to Havana by the United States Marine Hospital Service. The "Senator," the appropriate name of the vessel in question, has been completed in Philadelphia, and seems to be a most complete and up-to-date disinfecting plant. The value of such a vessel wherever ships have to be disinfected is unquestionable, for we can hardly doubt that much of the so-called "disinfection" to which ships are subjected is of a most imperfect character. By aid of such a vessel as the "Senator," however, it is possible not only to disinfect the passengers and their luggage in a much more perfect manner than can be done by any extemporised arrangements, but also to deal very thoroughly with any ship alongside of which it may be placed.

